# Adaptive metabolic rewiring and ion homeostasis enhance oxidative stress resistance in *Lacticaseibacillus casei*

**DOI:** 10.1128/aem.01854-25

**Published:** 2025-12-16

**Authors:** Lei Su, Ruisi Yang, Shan Li, Qilin Zhang, Feng Wang, Lianbing Lin, Yicen Lin

**Affiliations:** 1 Faculty of Life Science and Technology, Kunming University of Science and Technology 47910, Kunming, Yunnan, China; 2 Engineering Research Center for Replacement Technology of Feed Antibiotics of Yunnan College, Kunming, Yunnan, China; Universita degli Studi di Napoli Federico II, Portici, Italy

**Keywords:** *Lacticaseibacillus casei*, oxygen stress, adaptive evolution, ion homeostasis, transcriptomics

## Abstract

Enhancing the oxidative stress tolerance of microorganisms is critical for maintaining their viability and functionality in industrial fermentation under aerobic conditions. The present study used adaptive laboratory evolution to enhance the oxidative stress resistance of *Lacticaseibacillus casei*, a widely used lactic acid bacterium (LAB) in fermentation. By applying serial propagations with batch cultures under high oxygen conditions, the evolved strain exhibited enhanced H_2_O_2_ tolerance, improved metal ion chelation, and increased biofilm formation. By combining whole genome sequencing and re-sequencing, these phenotypic changes were attributed to key mutations involved in cell structure and metal ion homeostasis. Moreover, transcriptomic and metabolomic analyses revealed that, although both wild type and evolved strains employed a conserved stringent response by repressing *de novo* purine synthesis, the evolved strain adopted additional mechanisms to achieve this. These included enhanced purine salvage to support nucleic acid synthesis, increased transmembrane transport to improve carbon utilization, and upregulated citrate metabolism to generate proton motive force and maintain intracellular redox balance. Furthermore, strengthened copper ion homeostasis helped mitigate Fenton reaction–induced oxidative damage. These findings provide new insights into molecular adaptation and suggest a practical strategy to improve probiotic robustness in fermented foods.

**IMPORTANCE:**

Enhancing the oxidative stress tolerance of microorganisms is critical for maintaining their viability and functionality in industrial fermentation under aerobic conditions. This study demonstrates a non-GMO approach using adaptive laboratory evolution to enhance the robustness of *Lacticaseibacillus casei*. By integrating multiple characterization methods and combining genomics, transcriptomics, and metabolomics, the results revealed that the evolved strain developed significantly improved hydrogen peroxide resistance through key phenotypic and molecular adaptations. These findings uncover adaptive strategies in lactic acid bacteria and provide practical insights for optimizing probiotic performance in food and health applications without genetic engineering.

## INTRODUCTION

Lactic acid bacteria (LAB), including *Lacticaseibacillus casei*, are Gram-positive microorganisms with fermentative metabolism that are oxygen-tolerant and widely used in fermentation. Although they primarily rely on anaerobic fermentation, oxygen has been shown to enhance their growth and energy metabolism. Aerobic growth and respiration may confer both technological and physiological advantages on LAB strains (1–3). In *Lactiplantibacillus plantarum*, respiration enhanced growth and antioxidant capacity and improved survival during low-temperature storage (4). Some LAB isolates can significantly enhance their energy yield via aerobic respiration or partially oxidative metabolic pathways (5, 6). This energy advantage facilitates the survival and proliferation of LAB in competitive ecological niches, such as the gut, and their oxygen-tolerant phenotypes are advantageous in food industry applications (7). Oxygen metabolism inevitably produces reactive oxygen species (ROS), such as superoxide anion (O_2_⁻), hydrogen peroxide (H_2_O_2_), and hydroxyl radicals (OH·), which cause oxidative stress by damaging proteins, lipids, and DNA (8). To counteract this, LAB have evolved diverse antioxidant defenses, including enzymes (e.g., superoxide dismutase [SOD], peroxidases), non-enzymatic antioxidants (e.g., glutathione [GSH]), and regulatory systems that mitigate oxidative stress (3). Among these ROS, H_2_O_2_ is a major contributor due to its stability, membrane permeability, capacity to generate OH· via the Fenton reaction, and dual role as both a signaling molecule and a toxin (9).

Beyond producing detoxifying compounds, LAB fundamentally reprogram metabolism, such as modulating glycolysis and the fate of pyruvate, to adapt to diverse stressors. Multi-omics approaches are widely employed to elucidate metabolic reprogramming during stress responses (10). For example, under H_2_O_2_ stress, three LAB strains showed mostly active responses in membrane remodeling and metabolic pathways to combat ROS (11). Metabolomic profiling of *Pediococcus pentosaceus* isolated from dry sausage revealed that its protection against oxidative stress is associated with elevated amino acid levels and carbohydrate accumulation (12). Under oxidative stress, LAB showed metabolic shifts in carbohydrates, amino acids, and fatty acids, along with cell elongation or shortening and membrane thickness changes (13). Altered membrane fatty acid composition, especially increased unsaturated fatty acids, enhances stress resistance in pre-adapted LAB cells (14). Also, LAB maintain intracellular redox balance by exporting toxic reactive metals and preventing Fenton reaction-induced oxidative stress (15, 16). HprA1 protein confers hydrogen peroxide resistance in *L. casei* strain Shirota, which binds intracellular iron and prevents hydroxyl radical formation (17). Existing studies have primarily focused on these different responses to ROS stress, while how LAB enhance their tolerance and the underlying regulatory networks remains largely unknown.

Adaptive laboratory evolution (ALE) is a powerful approach for enhancing the adaptability of industrially relevant microorganisms, which avoids direct, targeted gene manipulation (18). Using ALE combined with whole-genome sequencing, adaptation to an aerobic lifestyle may serve as a natural strategy for developing LAB strains with enhanced oxygen consumption capacity and oxidative stress tolerance (19). The improved traits are beneficial for developing robust strains and reducing oxidative reactions in food products (20). During the process, the thioredoxin reductase system is a redox mechanism essential for aerobic growth (21). Investigating the regulatory factors and adaptive mechanisms underlying oxygen tolerance in LAB may help identify novel oxygen-tolerant phenotypes and enhance the fitness and competitiveness of starter cultures and probiotics (22). We previously demonstrated that enhanced antioxidant capacity improves fermentation performance and the viability of *L. casei* during cold storage (23); however, the underlying molecular mechanisms remain unclear. In this study, we selected a laboratory-isolated strain of *L. casei* R1 from blueberry fermented products, which possesses the thioredoxin reductase system, and subjected it to aerobic adaptation using ALE. Phenotypic mapping and multi-omics analyses were then integrated to elucidate the evolutionary molecular mechanisms underlying the enhanced anti-oxidative traits of evolved strain compared with the wild type.

## MATERIALS AND METHODS

### Bacterial strains and cultivation conditions

*L. casei R1* was originally isolated from an enzyme preparation derived from a fermented blueberry product at the Yunnan Provincial Engineering Research Center for Feed Antibiotic Alternatives, Kunming University of Science and Technology. The wild-type *L. casei* R1 (CGMCC No. 29492) was deposited at the China General Microbiological Culture Collection Center, under the Institute of Microbiology, Chinese Academy of Sciences. ALE was performed under controlled dissolved oxygen (DO) conditions by oxygen supplementation, following established bacterial cultivation methods (24). *L. casei* was initially inoculated at 2% (v/v, 2 mL) into 100 mL of MRS broth in 250 mL Erlenmeyer flasks and incubated at 37°C with shaking at 120 rpm. After 16 h of cultivation, 1 mL of culture was serially diluted and spread onto MRS agar plates, which were air-dried and incubated inverted at 37°C for 24 h. Single colonies were then selected from the MRS agar plates and subsequently propagated for the evolution experiments. *L. casei* was cultured under high and low DO conditions by adjusting fermenter headspace volumes to 30% and 2% (3.42–4.87 mg/L; 0.41–0.65 mg/L), respectively. DO levels were regulated by replacing the headspace air using a BioFlo 120 bioreactor system. After 24 h of incubation (37°C, initial pH 6.2, shaken at 120 rpm), a 2% aliquot was transferred to fresh medium, and the adaptive evolution process was continued for 18 successive batch transfers. As demonstrated in our previous work (23), intermediate rounds (3–15) exhibited gradual improvement, whereas by round 18, the population had reached a plateau in growth and H_2_O_2_ tolerance. The MRS liquid medium was prepared with the following components (g/L): 10.0 g peptone, 5.0 g beef extract, 4.0 g yeast extract, 20.0 g glucose, 1.0 mL Tween-80, 2.0 g K_2_HPO_4_, 5.0 g sodium acetate, 2.0 g ammonium citrate, 0.2 g MgSO_4_·7H_2_O, and 0.05 g MnSO_4_·4H_2_O, and the final pH was adjusted to 6.2 ± 0.2. MRS solid medium contained the same components as the liquid formulation with the addition of 15.0 g/L agar.

Evolved strains from the 18th round of aerated cultivation were plated for colony analysis. To verify the purity of the evolved strains, three single colonies (designated EvoA, EvoB, and EvoC) were randomly picked from the evolved strain plates. The 16S rRNA genes were amplified using universal primers: 27F and 1492R (27F: 5′-AGAGTTTGATCMTGGCTCAG-3′; 1492R: 5′-TACGGYTACCTTGTTACGACTT-3′). Cycling conditions: initial denaturation 95°C for 3 min; 30 cycles of 95°C for 30 s, 55°C for 30 s, 72°C for 90 s; final extension 72°C for 5 min. Amplified fragments were purified by kit (ExoSAP-IT) and sent to Sangon Biotech (Shanghai, China) for Sanger sequencing. The consensus sequence was queried against the GenBank database using BLASTn to determine the closest matches. These 16S rRNA sequences were generated solely to verify the absence of contamination; they are not part of the primary data set and were not uploaded to a public database.

The biomass accumulation of the evolved isolates EvoA, EvoB, and EvoC was compared with that of the wild-type strain under aerated and non-aerated conditions. At 16 h, cultures were diluted and plated on MRS agar to facilitate comparison of morphology. Bacterial biomass was assessed by measuring optical density at 600 nm (OD_600_) using a Varioskan LUX multimode microplate reader (Thermo Fisher Scientific), with OD_600_ values positively correlating with cell concentration.

### Assessment of hydrogen peroxide tolerance

Resistance testing was performed using the previously described method (25). Bacteria were pre-cultured in MRS broth for 16 h and then inoculated at 2% (v/v) into MRS broth containing 0.75 and 1.5 mM hydrogen peroxide for 8 h of cultivation. Bacterial growth was measured at 600 nm using a Varioskan LUX Multimode Microplate Reader (Thermo Fisher Scientific), and results were expressed as OD_600_. All measurements were performed in triplicate. The proportion of viable cells in the wild-type and evolved strains under 0.75 and 1.5 mM hydrogen peroxide stress was assessed by flow cytometry using the Cell-Check bacterial viability kit (A018, ABP Biosciences). Samples were diluted in 0.85% NaCl to ~10^6^ CFU/mL. Then, 0.3 µL of propidium iodide was added to 200 µL of each sample, followed by 15-min incubation in the dark. Analysis was performed using an air-cooled argon laser (excitation: 488 nm; emission >630 nm). Unstained samples served as controls. Measurements were performed in triplicate using a BD LSR Fortessa cell analyzer.

### Metal binding and antioxidant activity test

Bacteria were inoculated at 2% (v/v) into 100 mL MRS broth and cultivated at 37°C with shaking at 120 rpm for 16 h under either non-stress conditions or with 1.5 mM hydrogen peroxide. The cell density was adjusted to 10^6^ CFU/mL, and the cells were disrupted on ice by sonication (300 W, 3 s pulses with 7 s intervals, total 3 min). The lysates were centrifuged at 12,000 × *g* for 10 min at 4°C, and the resulting supernatants were collected as intracellular extracts for subsequent analysis. All measurements were normalized to 10^6^ CFU/mL to allow direct comparison between strains.

Excess intracellular free iron generates ROS via the Fenton reaction, leading to oxidative damage; thus, intracellular metal ion chelation confers resistance to oxidative stress in evolved LAB. Iron chelation activity was assessed as previously described (26). Briefly, 0.5 mL of intracellular extract was mixed with 0.1 mL of ascorbic acid (1 g/L), 0.1 mL of FeSO_4_ (0.4 g/L), and 1 mL of NaOH (0.2 M). The mixture was incubated at 37°C, followed by the addition of 0.2 mL of TCA (10%) and an additional 20-min incubation. After centrifugation, the supernatant was mixed with 0.5 mL of *o*-phenanthroline (1 g/L). Absorbance was measured at 510 nm after 10 min of reaction. All measurements were performed in triplicate.

Chelation activity was assessed using *o*-phenanthroline purple as a chromogenic agent, as described (27). A total of 50 µL hydrolysate (1 mg/mL) was added to a solution containing 250 µL of 50 mM sodium acetate buffer (pH 6.0), 6.25 µL of 4 mM o-phenanthroline purple, and 1 µg Cu^2+^ (as CuSO_4_). The reaction was incubated at room temperature for 1 min. Unbound Cu^2+^ reacted with o-phenanthroline purple, forming a chromogenic complex detectable at 632 nm. Chelator-induced dissociation of the complex caused a color shift from purple to yellow. Chelation activity was quantified by the decrease in absorbance at 632 nm.

Overnight cultures (16 h; 37°C) of the wild-type and evolved strains (10^6^ CFU/mL) were used to evaluate reduced glutathione (GSH), glutathione peroxidase (GSH-Px) activity (Grace, Suzhou, China), and NADH oxidase (NOX) activity (BOXBIO, China). GSH reacts with DTNB to form a yellow compound with peak absorbance at 412 nm. GSH-Px catalyzes the oxidation of GSH by cumene hydroperoxide (Cum-OOH), resulting in decreased GSH levels. Reduced absorbance at 412 nm reflects higher GSH-Px activity. NOX converts NADH to NAD^+^ and reduces 2,6-dichlorophenolindophenol (DCPIP) from blue to colorless. DCPIP reduction was monitored at 600 nm to quantify NOX activity.

### Morphological responses of wild-type and evolved strains to oxidative stress

Scanning electron microscopy (SEM) was conducted using a Regulus 8220 microscope (Hitachi, Tokyo, Japan). Bacterial cells were exposed to 1.5 mM hydrogen peroxide at 37°C with shaking for 3 h, pelleted by centrifugation, and fixed overnight at 4°C in 2.5% freshly prepared glutaraldehyde. Samples were dehydrated through a graded ethanol series (50%, 70%, 85%, 95%, and 100%; 15 min per step, with 100% ethanol repeated three times). Dehydrated cells were subjected to critical point drying using liquid CO_2_, with isoamyl acetate as a transitional fluid. Temperature and pressure were gradually increased to the CO_2_ critical point (31.1°C, 7.38 MPa) and then slowly released to preserve structural integrity. Samples were dried, mounted, cleaned, and sputter-coated with a 15–20 nm gold layer using a Hitachi MC1000 sputter coater under Ar plasma, followed by SEM imaging.

Transmission electron microscopy (TEM) was performed on cell pellets dehydrated through a graded ethanol (30%–100%) and propylene oxide series. Samples were infiltrated with a 1:1 (v/v) mixture of propylene oxide and epoxy resin, embedded in pure epoxy resin, and sectioned (~70 nm) using an ultramicrotome (UC-7, Leica). Ultrathin sections were stained with 2% uranyl acetate and lead citrate and imaged with a transmission electron microscope (HT7800, Hitachi, Japan). Cell wall thickness was quantified from TEM images using ImageJ software.

### Biofilm formation analysis

Confocal laser scanning microscopy (CLSM) was used to assess biofilm formation by the evolved and wild-type strains. One milliliter of overnight culture (16 h) was centrifuged (5,000 × *g* for 5 min), the supernatant was removed, and the bacterial pellet was resuspended in 1 mL TSB for later use. Bacteria were cultured in TSB medium with a 2% (v/v) inoculum and incubated statically for 16 h, and the glass slides were washed twice with PBS buffer after biofilm formation. Live and dead cells were stained at room temperature with 1 µM SYTO9 and propidium iodide (PI) for 20 min in the dark. After staining, slides were washed twice with PBS (1 mL) and observed using an SP8 (Leica, Germany) with a 20× objective lens and standard filters. The TSB liquid medium contained was prepared with the following components (g/L): tryptone 17.0, soy peptone 3.0, K_2_HPO_4_ 2.5, NaCl 5.0, and glucose 2.5, with a final pH of 7.3 ± 0.2. PBS was prepared by dissolving NaCl (8.0 g/L), KCl (0.2 g/L), Na_2_HPO_4_ (1.44 g/L), and KH_2_PO_4_ (0.24 g/L) in distilled water to a final volume of 1,000 mL.

Crystal violet was used to quantify biofilm formation in wild-type and evolved strains, following the method as described with modifications (28). Bacterial cultures were grown to the exponential phase (16 h), harvested by centrifugation at 5,000 × *g* for 5 min at room temperature, and resuspended in TSB medium to a final concentration of approximately 10^6^ colony-forming units (CFU)/mL. Briefly, 2% wild-type and evolved strains (approximately 10^6^ CFU/mL) were inoculated into 96-well plates by adding 4 µL of bacterial suspension to 196 µL of TSB medium with or without 1.5 mM hydrogen peroxide. After 2 days of cultivation, the bacterial culture was used to determine the number of viable cells by CFU counting. The wells were then washed three times with sterile water (200 µL) and air-dried at room temperature. Next, 0.1% crystal violet solution was added to each well and incubated at room temperature for 30 min. The staining solution was discarded, and the wells were rinsed with distilled water to remove any residual dye. After air-drying, 95% ethanol was added to each well and incubated at room temperature for 30 min to decolorize. Finally, absorbance at 570 nm was measured using a Varioskan LUX Multimode Microplate Reader (Thermo Fisher Scientific).

### Whole-genome sequencing and variant calling

Whole-genome sequencing was first conducted on the wild type strain to establish a reference genome. Subsequently, single nucleotide polymorphisms (SNPs) and other genetic variations were identified by comparing the genomes of three evolved strains (EvoA, EvoB, and EvoC) against the wild-type reference. Specifically, three biological replicates were performed for each of the above three randomly selected evolved clones, together with the wild type. Key mutations were further analyzed to identify genes potentially associated with the evolved phenotypes. Genomic DNA was extracted using the Wizard Genomic DNA Purification Kit (Promega, Madison, WI, USA). Whole-genome sequencing was performed using a combination of the PacBio RS II Single Molecule Real-Time (SMRT) and Illumina platforms. Illumina libraries were prepared using the NEXTflex Rapid DNA-Seq Kit from sheared DNA fragments. The libraries were then subjected to paired-end sequencing (2 × 150 bp) on the Illumina HiSeq X Ten platform. Data from both PacBio and Illumina platforms were used for bioinformatic analysis.

### Transcriptomic analysis

The wild-type and evolved strains were cultured overnight in MRS medium for approximately 16 h to reach the exponential phase, exposed to 1.5 mM hydrogen peroxide for 2 h, and subsequently sent to Shanghai Majorbio Bio-Pharm Tech (Shanghai, China) for RNA extraction and sequencing analysis. The raw data were subjected to a series of preprocessing steps to obtain high-quality reads, including the removal of adapter sequences and low-quality bases. RNA integrity was assessed using the Agilent 5300 Fragment Analyzer. The high-quality reads were aligned to the reference genome sequence using Bowtie 2.0. PCA and Venn analysis were performed to cluster the samples, identify influential samples, and determine co-expressed genes. To identify differentially expressed genes (DEGs) between the wild-type and evolved strains, RSEM was used to calculate transcript expression with a threshold of |log2FC| ≥ 1 and p-adjust < 0.05. GO enrichment analysis was then performed to classify DEGs into Biological Process, Cellular Component, and Molecular Function categories, while metabolic pathways were analyzed using KEGG and iPath. Functional annotation was conducted against databases of NR, Swiss-Prot, Pfam, COG, GO, KEGG, CAZy, CARD, and VFDB. GO enrichment was carried out with Goatools using Fisher’s exact test, with all annotated genes as background and significance defined at FDR-adjusted *P* < 0.05 (29).

### Metabolomic analysis

To investigate intracellular metabolic differences between the wild-type and evolved strains under oxidative stress, overnight cultures were first grown to the exponential phase (16 h; 37°C), after which, cells were collected by centrifugation, treated with 1.5 mM hydrogen peroxide, and incubated statically at 37°C for 2 h prior to omics analyses. Metabolomic profiling was then performed using liquid chromatography–mass spectrometry (LC–MS), following a previously reported protocol (30). In brief, the workflow for intracellular metabolite analysis included sampling, quenching, extraction, detection, and data analysis. Each treatment group included six biological replicates. Peak picking and retention time alignment were carried out using XCMS. Metabolite identification was performed using the metaX package, based on matches to KEGG and HMDB databases. Metabolite annotation was further supported by an in-house tandem MS (MS/MS) spectral library from LC Science Co., Ltd. Features detected in fewer than 50% of QC samples or 80% of biological samples were excluded. Differences in metabolite concentrations between treatment groups were assessed using Student’s *t*-test with Benjamini–Hochberg correction. Partial least squares discriminant analysis (PLS-DA) and orthogonal projections to latent structures discriminant analysis (OPLS-DA) were employed to identify metabolites that distinguished between the two treatments. Metabolites with |log_2_(fold change)| > 1, variable importance in projection (VIP) > 1, and *P*-value < 0.05 were considered significantly different (10). The metabolomics data generated in this study have been deposited in the Metabolomics Workbench under Study ID PR002633.

### Statistical analysis

Transcriptomic analyses were performed with three biological replicates, whereas metabolomic analyses were conducted with six biological replicates. All other experiments were also performed using three biological replicates. Data are presented as mean ± standard error of the mean (SEM). Statistical analysis was conducted using one-way ANOVA for comparisons among multiple groups and unpaired *t*-tests for comparisons between two groups. GraphPad Prism 8 (San Diego, CA, USA) was used for statistical testing, while data visualization was performed using the R programming language.

## RESULTS

### Laboratory evolution leads to improved tolerance of hydrogen peroxide

**Fig 1:**
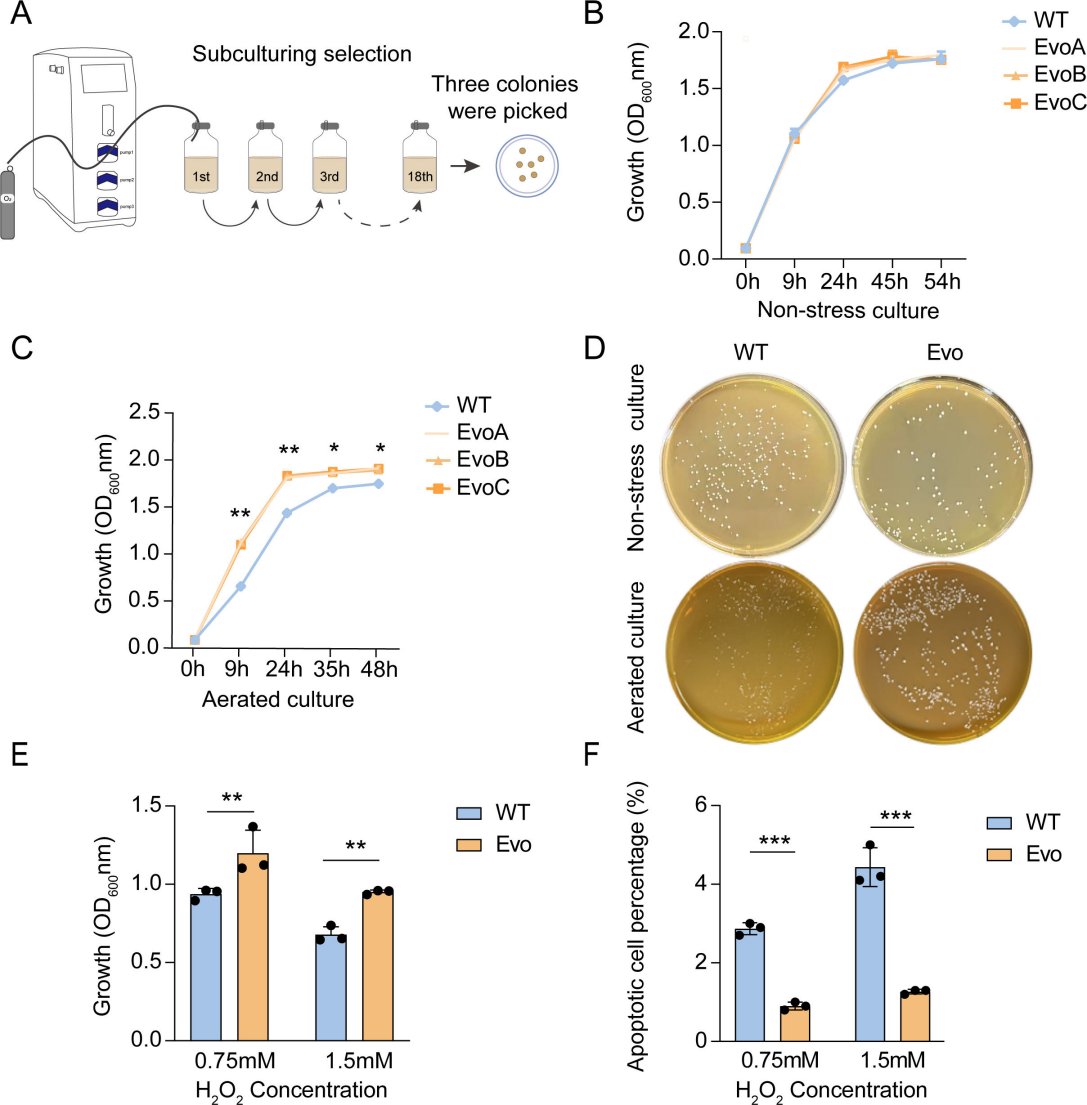
Aerobic adaptive evolution of *L. casei* and phenotypic characterization of wild type and evolved strains. (**A**) Eighteen rounds of aerobic adaptive evolution of *L. casei* under aeration stress. (**B**) Biomass of the wild type (WT) and three evolved isolates (EvoA, EvoB, EvoC) under non-stress culture. (**C**) Biomass of the WT and three evolved isolates (EvoA, EvoB, EvoC) under aerated stressed culture. (**D**) Colony morphology of the WT and evolved strain (EvoC was designated as Evo) under non-stress and aerated conditions. (**E**) Biomass of the WT and Evo under hydrogen peroxide stress. (**F**) Apoptotic cells of the WT and Evo under hydrogen peroxide stress measured by flow cytometry. Values are shown as mean ± SD (*n* = 3). *P < 0.05; ** *P* < 0.01; ***P < 0.001; two-tailed t test.

After adaptive evolution under oxygen stress (Fig. 1A), colony formation and ROS tolerance were evaluated. Three evolved single colonies (EvoA, EvoB, and EvoC) were randomly selected and confirmed as *L. casei* through comparison with the NCBI database. Growth characteristics were compared under aerated culture conditions. Under non-stress conditions, three evolved single colonies (EvoA, EvoB, and EvoC) showed biomass accumulation comparable to that of the wild type, with no significant differences (Fig. 1B). However, after 48 h under aerated cultivation (with ROS stress), EvoA, EvoB, and EvoC accumulated more biomass than the wild type (*P* < 0.05), with no significant differences among the three evolved strains (Fig. 1C). Since the three randomly selected evolved strains exhibited similar phenotypes, we chose one clonal isolate (EvoC) as the representative of the evolved population for subsequent experiments. Colony morphology was assessed on agar plates. Under non-stress conditions, the wild-type and evolved strain (EvoC) exhibited comparable colony sizes. However, under aerated conditions, the wild type formed smaller colonies, whereas the evolved strains produced significantly larger ones (Fig. 1D).

Next, ROS tolerance of the evolved strain was assessed under both low (0.75 mM) and high concentrations (1.5 mM) of hydrogen peroxide. The evolved strains accumulated significantly more biomass than the wild-type strain (Fig. 1E). Flow cytometry was subsequently conducted to assess the proportion of viable cells under oxidative stress (Fig. 1F). The results showed that evolved strains consistently exhibited a higher percentage of viable cells than the wild type, indicating that they possessed enhanced survival rates under ROS stress after adaptive evolution.

### Metal chelation and antioxidant enzyme activity contribute to enhanced oxidative resistance in evolved strains

**Fig 2:**
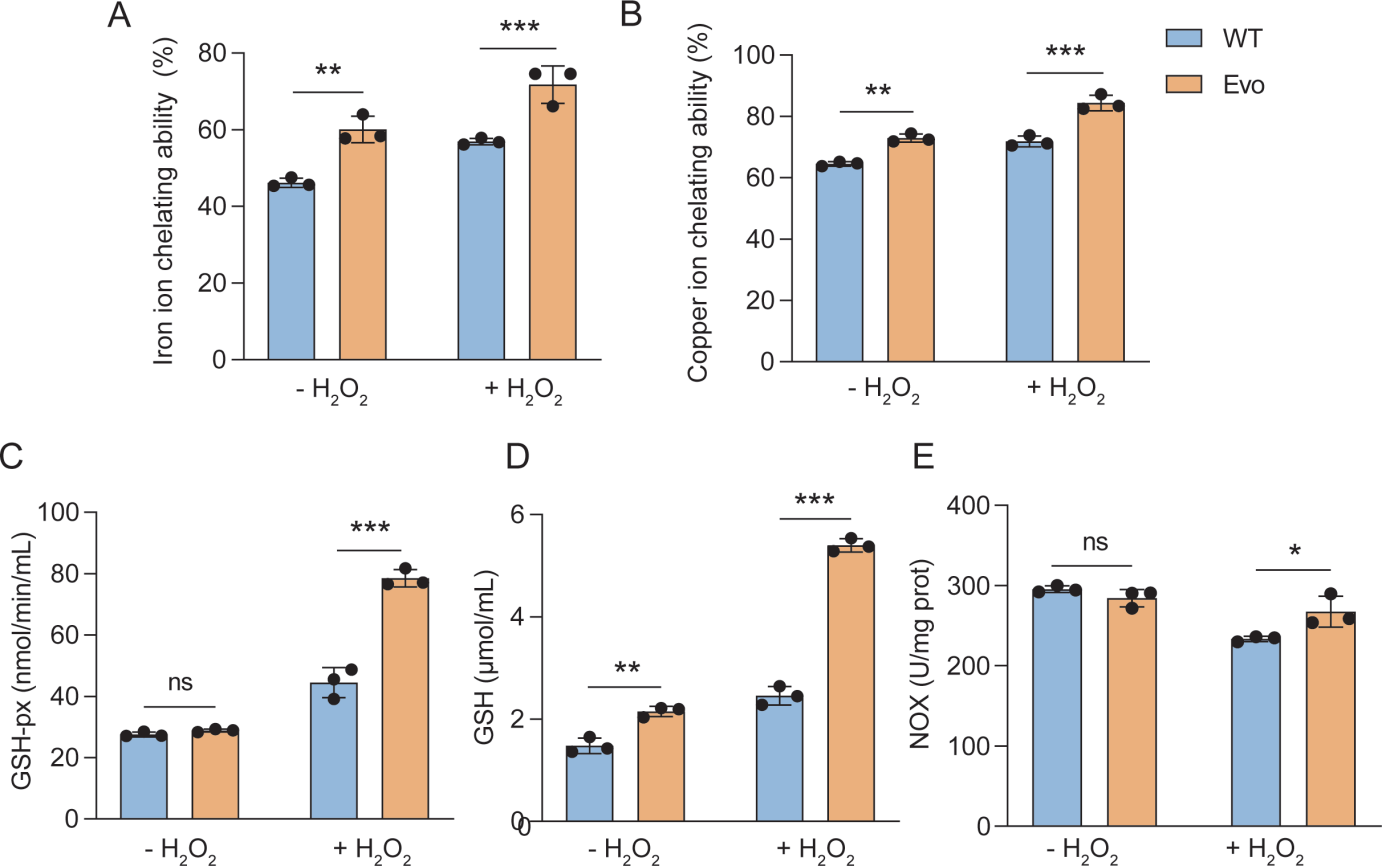
Metal-chelating and antioxidant properties of wild type and evolved *L. casei* strains. (**A**) Fe^2+^ chelation capacity (%) under non-stress and 1.5 mM H_2_O_2_-stress conditions. (**B**) Cu^2+^ chelation capacity (%) under non-stress and 1.5 mM H_2_O_2_-stress conditions. (**C**) GSH-Px enzymatic activity under non-stress and 1.5 mM H_2_O_2_-stress conditions. (**D**) GSH concentration under non-stress and 1.5 mM H_2_O_2_-stress conditions. (**E**) NOX enzymatic activity under non-stress and 1.5 mM H_2_O_2_ stress conditions. The test cultures were obtained from the same overnight culture (16 h, 10^6^ CFU/mL cells). “- H_2_O_2_” indicates non-stress cultivation, and “+ H_2_O_2_” indicates exposure to 1.5 mM H_2_O_2_ stress for 2 h. ns, not significant; Values are shown as mean ± SD (*n* = 3). *P < 0.05; **P < 0.01; ***P < 0.001; two-tailed *t* test.

Metal ions, such as Fe^2+^, can induce lipid peroxidation, triggering a cascade reaction that leads to the generation of peroxyl and alkoxy radicals. As shown in Fig. 2A and B, the evolved strain exhibited significantly greater Fe^2+^ and Cu^2+^ chelation capacities than the wild type under both non-stress and oxidative stress conditions, suggesting an intrinsic enhancement of non-enzymatic antioxidant defense.

Under non-stress conditions, no difference in GSH-Px activity was observed between the two strains. However, under hydrogen peroxide stress, GSH-Px activity in the evolved strain (Fig. 2C) was significantly higher than that in the wild type, indicating that enzymatic antioxidant systems were more inducible in the evolved background. In addition, the evolved strain maintained a significantly higher intracellular GSH content under both non-stress and oxidative stress conditions (Fig. 2D), which likely provides a metabolic reservoir for detoxification. NOX activity showed a different pattern: while no difference was detected under non-stress conditions, the evolved strain displayed markedly higher activity under oxidative stress (Fig. 2E), consistent with enhanced ROS detoxification capacity. Together, these results indicate that the evolved strain developed both constitutive and inducible antioxidant advantages compared with the wild type, which may underlie its superior tolerance to oxidative stress.

### Improved morphological stability under oxidative stress in evolved strains

**Fig 3:**
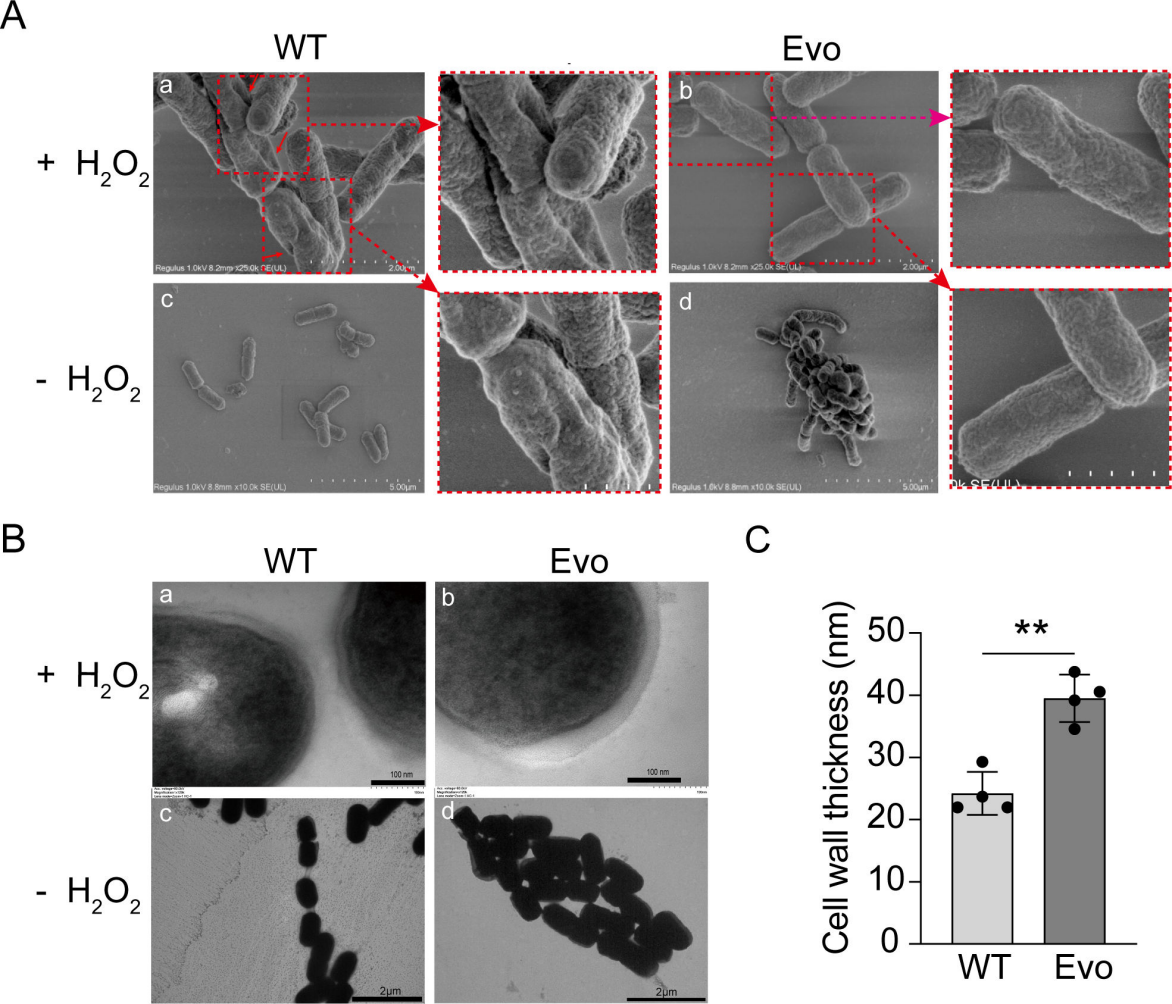
Electron microscopy analysis of wild type and evolved *L. casei* strains. (**A**) Scanning electron microscopy images. (**B**) TEM images. “- H_2_O_2_” indicates non-stress cultivation in MRS broth, and “+ H_2_O_2_” indicates exposure to 1.5 mM H_2_O_2_ stress in MRS broth for 2 h. (**C**) Quantification of cell wall thickness. Images were analyzed using ImageJ software. Values are shown as mean ± SD (*n* = 4). **P < 0.01; two-tailed t test.

Scanning electron microscopy (SEM) was employed to assess morphological alterations of the wild-type and evolved strains under hydrogen peroxide stress. As shown in Fig. 3A, exposure of the wild-type strain to 1.5 mM hydrogen peroxide resulted in pronounced structural abnormalities, including disrupted cell division and surface deformation (Fig. 3Aa). In contrast, the evolved strain maintained a more intact and compact morphology under the same conditions (Fig. 3Ab). These observations suggest that hydrogen peroxide stress induces severe morphological and structural damage in the *L. casei* R1 wild type, whereas the evolved strain displays enhanced structural stability and stress tolerance. Under non-stress conditions, both the wild-type (Fig. 3Ac) and evolved strain (Fig. 3Ad) exhibited normal morphology.

TEM further highlighted structural differences in cell envelope integrity. The wild-type strain exhibited a relatively thin cell wall (Fig. 3Ba), whereas the evolved strain displayed a markedly thicker and more robust cell wall (Fig. 3Bb). Quantitative analysis confirmed that the evolved strain possessed a significantly greater cell wall thickness than the wild type (*P* < 0.01, Fig. 3C). Under non-stress conditions, both the wild-type (Fig. 3Bc) and evolved strain (Fig. 3Bd) maintained normal ultrastructures, although the thicker cell wall phenotype remained evident in the evolved strain. Collectively, these SEM and TEM analyses demonstrate that the evolved strain achieves superior structural resilience through cell wall fortification, providing a critical morphological basis for its improved oxidative stress tolerance.

### Increased biofilm-forming ability in evolved strains

**Fig 4:**
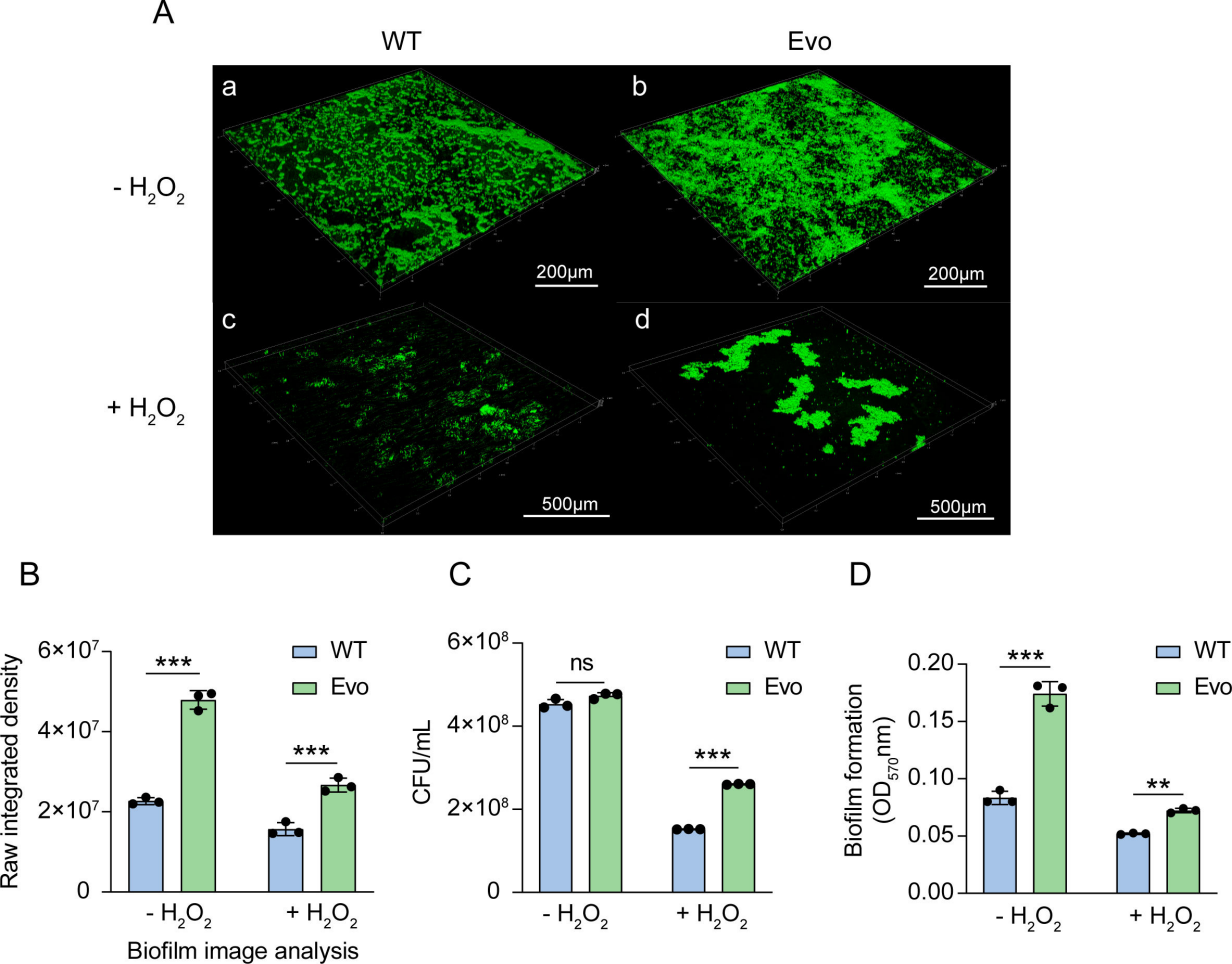
Biofilm formation and biomass comparison of wild type and evolved *L. casei* strains under non-stress and stress conditions. (**A**) CLSM biofilm imaging of wild-type and evolved strains. (**B**) Initial integrated density of biofilms formed by the wild-type and evolved strains. (**C**) Survival rates of the wild-type and evolved strains in TSB medium, a nutrient-rich medium containing peptides, amino acids, and sugars, with or without hydrogen peroxide stress. (**D**) Crystal violet assay of the wild-type and evolved strains under the presence or absence of H_2_O_2_ stress. Biofilm formation was assessed after 48 h of cultivation, using 1.5 mM H_2_O_2_ as the stressor. CLSM biofilm images were analyzed in ImageJ (Raw Integrated Density function), and the mean ± SD of three images per group was calculated. ns, not significant; **P < 0.01; ***P < 0.001; two-tailed t test.

Biofilm formation plays a crucial role in the environmental adaptability and stress tolerance in bacteria. To evaluate this trait, we performed confocal laser scanning microscopy (CLSM) analysis. As shown in Fig. 4A, the evolved strain (EvoC) exhibited enhanced biofilm formation both in the absence (Fig. 4Ab) and presence (Fig. 4Ad) of hydrogen peroxide, whereas the wild-type strain developed a less compact biofilm under oxidative stress (Fig. 4Ac). Quantitative assessment of biofilm formation using raw integrated density revealed that the evolved strains consistently exhibited significantly higher values than the wild type, regardless of stress conditions (Fig. 4B).

Biofilm formation was further quantified using the crystal violet assay. Under hydrogen peroxide stress, the evolved strains showed significantly higher survival rates than the wild type, while no substantial differences were observed under non-stress conditions (Fig. 4C). Consistently, in both the presence and absence of hydrogen peroxide, the evolved strains formed markedly more robust biofilms in TSB medium, a condition known to promote biofilm formation in LAB (Fig. 4D). Together, these results demonstrate that adaptive evolution enhanced the biofilm-forming capacity of the evolved strains, thereby contributing to their improved tolerance and survival under oxidative stress.

### Genomic analysis uncovers critical evolutionary changes

To elucidate the potential molecular mechanisms underlying increased oxygen tolerance, we sequenced the whole genome of *L. casei* R1 and performed variant calling analysis. The genome size of GMCC29492 is 3.09 Mb, with a GC content of 46.66%. A total of 3,420 protein-coding sequences were identified, along with 61 tRNA genes, 15 rRNA genes, and 1 tmRNA gene. Strain GMCC29492 harbors important antioxidant enzymes encoding genes, including NADH oxidase (*nox*) and NADH peroxidase (*npr*). In addition, genes related to thioredoxin (*Trx*)-thioredoxin reductase (*TrxR*) system were identified, including glutaredoxin (*nrdH*), thioredoxin (*trxA*), thioredoxin reductase (*trxB*), and thiol peroxidase (*tpx*). For the glutathione system, encoding genes of synthetase (*gsh*) and peroxidase (*bsaA*) were identified. These results provided genomic evidence for aerobic lifestyle and oxygen tolerance of GMCC29492.

Genome re-sequencing revealed 38 mutations. The mutations affected both coding and non-coding regions. Shared key mutations and corresponding amino acid sequence variations were identified in the three evolved isolates (EvoA, EvoB, and EvoC), each with three biological replicates (Table S1). Most mutations occurred in genes with known functions, including cell wall synthesis, protein secretion, transcriptional regulation, and stress response, suggesting that these mutations may influence the overall adaptability and environmental stress response capacity of the evolved strains. The most frequently mutated genes were transcriptional regulators (*nusA, nusG, mfd, yfbR, srrA, rcsC*). Another group of mutations related to iron homeostasis, including *feoA* (iron) and *mco* (copper). GO enrichment analysis revealed that mutated genes were significantly associated with intracellular protein-containing complexes (GO:0140535, *P* = 0.000319), most of which are related to transcriptional or post-transcriptional regulatory machinery.

### Transcriptomic profiling reveals conserved antioxidant responses in *L. casei*

**Fig 5:**
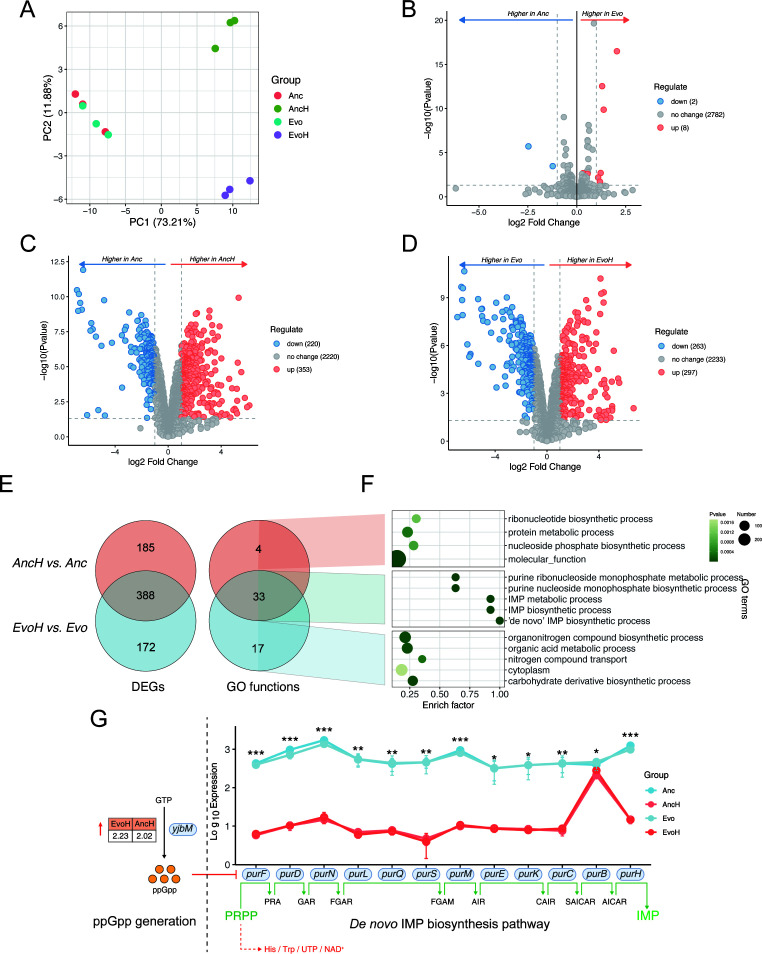
(**A**) PCA of transcripts from four groups. Anc and AncH: wild type cultures without and with stress; Evo and EvoH: evolved strain cultures without and with stress. (**B–D**) Volcano plot of differentially expressed genes in three comparisons. Evo vs Anc represents the comparison between evolved and wild-type transcriptomes under non-stress conditions. AncH vs Anc represents the comparison between stressed and non-stressed wild-type transcriptomes, and EvoH vs Evo represents the comparison between stressed and non-stressed evolved transcriptomes. (**E**) Venn diagram of differentially expressed genes and enriched GO pathways in two comparisons. (**F**) Shared and unique GO pathways of two comparisons. (**G**) Gene expression pattern of *de novo* IMP biosynthesis. ppGpp: guanosine 5-diphosphate 3-diphosphate; PRPP: phosphoribosyl pyrophosphate; PRA: phosphoribosylamine; GAR: glycinamide ribonucleotide; FGAR: formylglycinamide ribonucleotide; FGAM: formylglycinamidine ribonucleotide; AIR: aminoimidazole ribonucleotide; CAIR: carboxyaminoimidazole ribonucleotide; SAICAR: succinylaminoimidazole carboxamide ribonucleotide; AICAR: aminoimidazole carboxamide ribonucleotide; IMP: inosinemonophosphate. *P < 0.05; **P < 0.01; ***P < 0.001; Two-tailed *t* test. Error bars represent the standard deviation (SD), with data presented as mean ± SD, *n* = 3.

RNA-seq was used to identify the transcriptomes of the wild type strain and evolved strains with or without exposure to 1.5 mM hydrogen peroxide. RNA sample integrity and quality were assessed (Table S2). A total of 22,438,105 and 25,130,058 mean raw reads were obtained for the wild-type and evolved strain (EvoC), respectively. After removing contaminants and low-quality sequences, the remaining reads were mapped to the annotated genome. Principal component analysis (PCA) under different conditions (Fig. 5A) revealed similarities between the evolved and wild-type strains in the absence of H_2_O_2_ exposure. Results showed distinct gene expression patterns in the evolved strain when exposed to H_2_O_2_ compared with those from wild type.

DESeq2 was used to analyze the differentially expressed genes, which generated four pairwise comparisons. Under normal conditions, only eight DEGs were identified between the evolved and wild-type strains (Fig. 5B). Compared with the non-stress conditions, 573 and 560 DEGs were identified in the wild-type and evolved strains, respectively, after exposure (Fig. 5C and D). All DEGs across all comparisons are provided (Tables S3 to S6). This analysis highlights that the evolved strain possesses a distinct, inducible mechanism for resisting ROS, with no significant alteration in physiological activity under normal culture conditions. As shown in Fig. 5E, the two strains shared 388 DEGs in response to oxidative stress, while the wild-type and evolved strains uniquely expressed 185 and 172 DEGs, respectively. These findings suggest that transcriptional responses to ROS are largely conserved between the two strains.

Under stress, the wild type exhibited only four uniquely enriched GO pathways, primarily related to nucleotide and protein metabolism, suggesting limited unique responses. These pathways are typically associated with DNA/RNA damage, such as oxidative lesions and strand breaks (Fig. 5F). Significantly more GO terms (17) were enriched in the evolved strain under stress, predominantly related to substance metabolism and redox regulation (Table S7). These included pathways involving organic acid metabolism (e.g., carboxylic acids, oxoacids), nitrogen metabolism, carbohydrate derivatives, and redox homeostasis.

Most genes and GO terms reflected conserved mechanisms between the wild-type and evolved strains (Table S8). Thirty-three GO terms were significantly associated with purine metabolism, particularly the *de novo* IMP biosynthetic process (Fig. 5F). GTP pyrophosphokinase, which synthesizes (p)ppGpp, showed more than twofold upregulation in both strains under stress (Fig. 5G). These enzymes expressed in the *pur* operon are directly involved in the conversion of PRPP to IMP, and all involved genes were consistently downregulated in both strains under stress (Fig. 5G). Both the wild-type and evolved strains employed a conserved stringent response strategy—downregulating purine metabolism to sacrifice growth in favor of enhanced stress resistance.

### Integrated transcriptomic and metabolomic analyses reveal evolved strain-specific antioxidant adaptations

**Fig 6:**
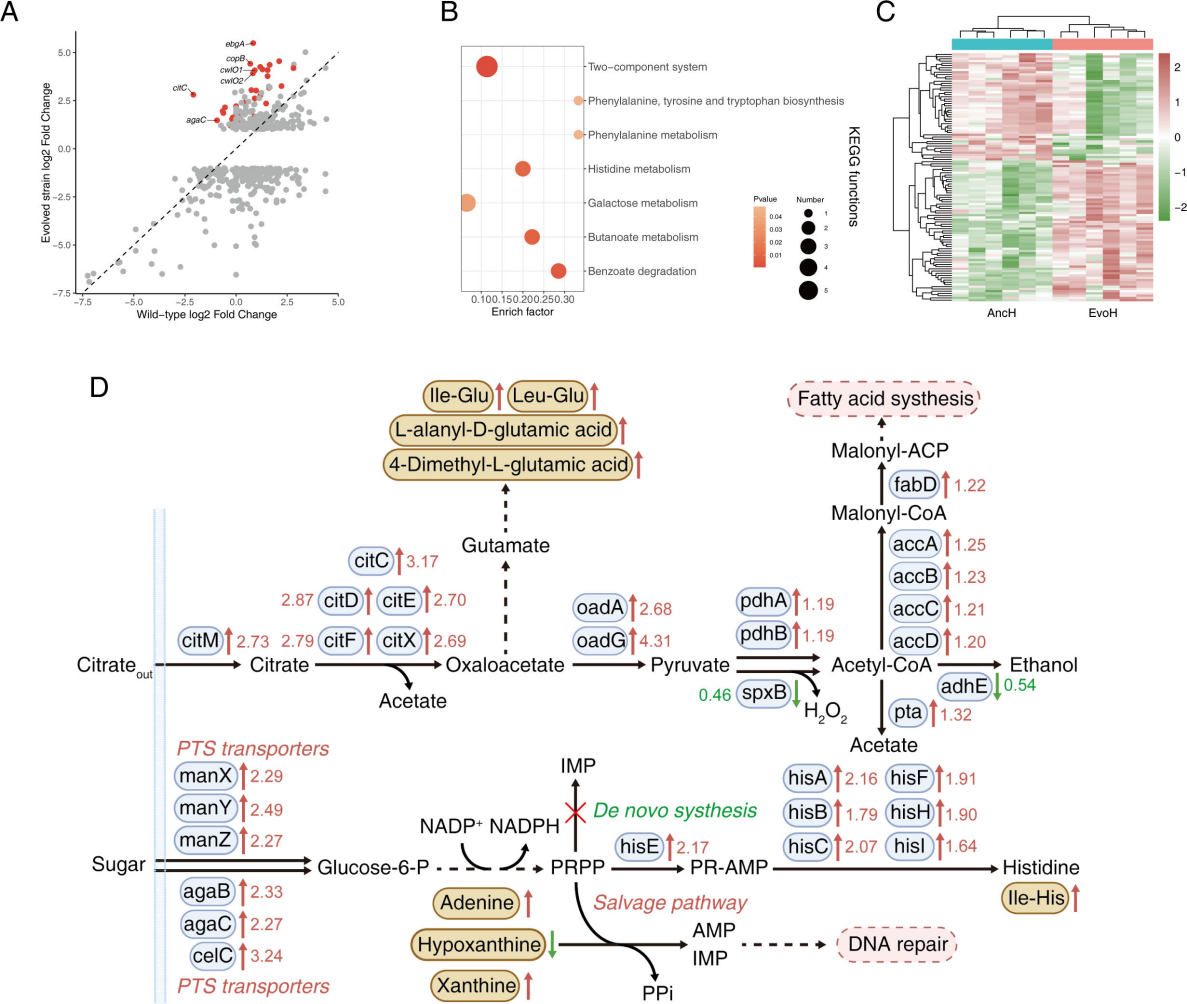
Integrated transcriptomic and metabolomic analysis. (**A**) Fold-change comparison of DEGs between the two strains under stress and non-stress conditions. (**B**) KEGG pathway enrichment analysis based on DEGs between wild-type and evolved strain under stress. (**C**) Heatmap of significantly altered metabolites under stress conditions comparing wild-type (AncH) and evolved strain (EvoH). (**D**) Gene pathway related to citrate and carbon metabolism. Blue ellipses represent significantly differentially expressed genes, yellow ellipses indicate significantly altered metabolites, and pink ellipses denote metabolic pathways. Solid lines represent direct biosynthetic relationships, while dashed lines indicate multi-step processes. Numbers represent fold change values. PPi: inorganic pyrophosphate; AMP: adenosine monophosphate; PR-AMP: phosphoribosyl-AMP; NADP^+^: nicotinamide adenine dinucleotide phosphate (oxidized form); NADPH: nicotinamide adenine dinucleotide phosphate.

Despite sharing conserved mechanisms with the wild type, the evolved strain was further analyzed through integrated transcriptomic and metabolomic profiling to reveal strain-specific enhancements in ROS adaptation. Differential expression analysis identified 40 significantly upregulated genes in the evolved strain compared with the wild type (Fig. S1; Table S9). Using the significantly DEGs in the evolved strain as a reference, we compared fold-change patterns in the wild type before and after stress, thereby identifying genes with pronounced expression differences (Fig. 6A). Results showed that the 40 genes significantly upregulated in the evolved strain maintained high fold changes and were mainly distributed on one side of the y-axis. *copB*, which encodes a copper-exporting ATPase, showed the highest upregulation, suggesting that the evolved strain mitigates oxidative damage by reducing intracellular copper levels to suppress Fenton reaction-derived ROS. CwlO encodes a cell wall hydrolase that is essential for maintaining cell morphology and remodeling the cell wall. Upregulation of the genes encoding cell wall hydrolases P40 (*cwlO1*) and C40 (*cwlO2*) suggests that the evolved strain may promote cellular elongation through cell wall remodeling, thereby facilitating division and proliferation.

KEGG enrichment analysis was performed on all significantly upregulated genes in the evolved strain (Fig. 6B). Within the two-component system, significant upregulation of a gene cluster involved in citrate metabolism—including *citM*, *citC*, *citD*, *citE*, *citF*, and *citX*—was observed. In addition, the evolved strain exhibited significant enrichment in pathways related to phenylalanine, tyrosine, and tryptophan biosynthesis, histidine metabolism, and phenylalanine degradation.

To investigate the metabolic shifts in the evolved strain under oxidative stress, intracellular metabolites were profiled using LC-MS. Twelve samples were analyzed via PLS-DA and OPLS-DA models (Fig. S2). Differential metabolites were identified based on the HMDB database, and relative abundance changes were visualized in a heatmap (Fig. 6C). A total of 111 differential metabolites were detected, of which 63 were upregulated. The top 20 differential metabolites are presented in Table S10. Several dipeptides associated with redox balance and antioxidant defense were enriched, including gamma-glutamylleucine and nicotinic acid, as well as a series of dipeptides such as lysine–isoleucine, leucine–lysine, isoleucine–aspartate, leucylglycine, and glycine–valine.

KEGG enrichment analysis revealed that metabolites related to purine metabolism were most significantly enriched (Fig. S3), including adenine, xanthine, and hypoxanthine. Changes in the abundance of these compounds suggest metabolic reprogramming to purine salvage pathway in the evolved strain. Significant enrichment of adenine may improve ATP synthesis efficiency, thereby enhancing metabolic activity and stress resistance. In addition, enrichment was observed in pathways related to fatty acid metabolism.

A metabolite–gene expression network revealed mechanisms by which the evolved strain enhances ROS tolerance through metabolic rewiring. This rewiring primarily involves citrate metabolism, fatty acid biosynthesis, purine metabolism, and PTS pathways, which are associated with enhanced regeneration of reducing power and ATP production (Fig. 6D). Notably, citrate metabolism was significantly upregulated, facilitating pyruvate production via the lyase–oxaloacetate decarboxylase (CL-OAD) pathway and increasing acetate output. Additionally, enhanced sugar uptake via PTS pathways is redirected toward the PPP to produce NADPH. Sustained PPP activity requires the regeneration of NADP^+^ through ROS-dependent oxidation. Beyond redox balancing, the PPP also provides essential biosynthetic precursors for nucleotide, amino acid, and lipid synthesis. In both the wild-type and evolved strains, *de novo* purine synthesis was suppressed (Fig. 6D). However, the purine salvage pathway, which was more efficient, was significantly upregulated in the evolved strain.

## DISCUSSION

The strain used in this study, *L. casei* R1, was taxonomically identified by whole-genome sequencing and deposited in the China General Microbiological Culture Collection Center (CGMCC No. 29492). Its safety and probiotic properties, including intestinal colonization, antioxidant activity, and inhibition of pathogens, have been previously reported (23). Following adaptive laboratory evolution, three single colonies exhibited consistent phenotypes under both aerated and non-aerated conditions, and one was selected as representative for further experiments. This suggests that the evolved populations were dominated by adaptively enhanced strains, allowing a representative isolate to be selected for further study (31). Under aeration, the evolved strains showed accelerated nutrient utilization and earlier biomass accumulation than the wild type, indicating distinct physiological adaptations to oxidative stress. When challenged with H_2_O_2_, the evolved strain exhibited enhanced survivability, indicating an improved tolerance to oxidative stress compared with the wild type. Together, these results demonstrate that our ALE strategy successfully yielded ROS-tolerant strains, providing a foundation for subsequent mechanistic investigations.

Excess intracellular free iron can trigger ROS production via the Fenton reaction, causing oxidative damage; hence, intracellular metal ion chelation represents an important non-enzymatic antioxidant defense in LAB (32). Previous studies have shown that probiotic LAB exhibit Fe^2+^ and Cu^2+^ chelation activities that help maintain redox homeostasis, as reported for *Lactococcus lactis* KCTC 3250, where antioxidant capacity was attributed mainly to metal chelation rather than SOD activity (33). As shown in Fig. 2A and B, the evolved strain exhibited significantly higher metal ion chelation capacity than the wild type, regardless of ROS presence. This suggests that its enhanced antioxidant ability is an inherent trait linked to genetic mutations. Given the non-enzymatic nature of this mechanism, such intrinsic enhancement may provide a competitive advantage over the wild type, as it avoids the metabolic cost of producing additional enzymes.

In addition to metal ion chelation, LAB protect against oxidative stress by scavenging ROS, reducing ascorbic acid auto-oxidation, and upregulating antioxidant enzymes such as catalase, peroxidase, and glutathione peroxidase (34). The GSH system (including GSH and GSH-Px) is particularly important for detoxifying H_2_O_2_, although some LAB species accumulate glutathione mainly through uptake rather than *de novo* synthesis (35). In the presence of ROS, the evolved strain showed significantly higher expression of this system than the wild type, suggesting that the associated enzymes may contribute to detoxification, consistent with previous reports (36). However, under non-stress conditions, GSH and GSH-Px levels differed between the two strains, indicating that GSH-Px is not constitutively upregulated. NOX may act in coordination with the GSH-Px system to alleviate oxidative stress (3). Although NOX contributes to ROS detoxification and oxygen consumption, its activity varies among strains and can decrease under excessive stress (37). As NOX reduces oxygen to hydrogen peroxide, an imbalance between NOX activity and GSH-Px capacity could influence ROS accumulation (38). In this study, the evolved strain exhibited higher NOX activity than the wild type under H_2_O_2_ stress, suggesting an enhanced ability to tolerate and survive oxidative challenge.

Strains with higher antioxidant enzyme activity show only mild surface damage under H_2_O_2_ stress, whereas those with lower activity suffer severe cell wall damage or lysis (11). The cell envelope and related genes are key for stress response (39), and surface shrinkage under oxidative stress has been linked to matrix metalloproteinase overexpression (40). LAB aggregation promotes intercellular communication, regulating metabolism, gene expression, and stress adaptability (41). Similar traits have been reported in *Lactobacillus curvatus* BSF206 and *Lactobacillus pentosus* AC1-2, which eliminate cariogenic bacteria in the oral cavity, inhibit pathogenic biofilm formation, and display tolerance to acid, bile salts, hydrogen peroxide, and lysozyme, thereby improving survival in the oral environment (42). Our previous work also showed that the evolved strain has excellent acid and bile salt resistance (23), indicating that its oxidative stress tolerance is part of a broader adaptation to harsh environments. Similar to the present study, cell adhesion and biofilm formation have been also identified as key factors in the stress resistance mechanisms of *Lactobacillus plantarum* (43). Biofilm formation not only promotes survival and colonization of probiotics under adverse environmental conditions but also enhances their stress resistance, providing significant support for their probiotic functions (44).

Genomic resequencing analysis revealed that *L. casei* inherently possesses a variety of antioxidant enzymes and systems that contribute to its oxidative resistance. During adaptive evolution, mutations were primarily enriched in transcriptional regulators. Such mutations can exert broad effects on bacterial gene expression, ultimately shaping metabolic pathways and stress responses. Among these, *srrA*, a respiratory response regulator, facilitates adaptation to hypoxic environments by modulating genes involved in anaerobic metabolism and virulence, thereby enhancing bacterial survival under oxygen-limited conditions (45). *srrA* has also been implicated in biofilm formation, suggesting a role in both stress adaptation and community behavior (46). In addition, mutations in genes related to metal ion homeostasis highlight their importance in oxidative stress adaptation, including *feoA* (iron) and *mco* (copper). In *Lactobacillus*, limiting intracellular iron accumulation is particularly critical, as it can reduce Fenton reaction–driven hydroxyl radical formation. Several metabolic genes were also affected. For instance, *hprK*, a regulatory kinase of the phosphotransferase system, is involved in carbohydrate uptake and metabolism (47). Gene *nox_2*, encoding NOX, contributes to maintaining redox balance in aerobic stress (48). Overall, these diverse genetic mutations collectively underpin the metabolic differences observed between evolved and wild-type strains. Nevertheless, further functional validation is required to confirm the specific roles of these candidate genes in oxidative stress resistance and adaptive metabolism.

In this study, a comprehensive comparison of gene expression between the evolved and wild-type strains under stress and non-stress conditions revealed adaptive molecular mechanisms. For example, genes involved in carbohydrate and organic acid metabolism were specifically upregulated in the evolved strain (Fig. 5F). Similarly, organic acid metabolism may enhance antioxidant capacity by producing protective compounds such as exopolysaccharides (49). Together, these metabolic shifts help offset ATP deficits caused by oxidative damage and contribute to improved stress tolerance in the evolved strain. In contrast, fewer unique GO functions were enriched in the wild type strain under stress (Fig. 5F), indicating a more limited adaptive response. Oxidative stress induces global metabolic reprogramming in *Lactobacillus* species, particularly in nucleotide and protein metabolism. ROS exposure commonly upregulates DNA repair and nucleotide biosynthesis enzymes (50), while in some cases, pyrimidine synthesis is suppressed to conserve resources (51). Protein metabolism is likewise remodeled, with increased amino acid biosynthesis, redox-sensitive enzymes, molecular chaperones, and proteases ensuring protein stability under stress (49, 52).

Furthermore, Fig. 5G highlights the activation of a conserved antioxidant mechanism, the stringent response, in both evolved and wild-type strains. Under stress conditions, LAB typically adopt a stringent response, downregulating ribosome biogenesis, translation, and post-translational processes to enhance survival at the expense of growth (10). The stringent response is known to suppress GTP synthesis, thereby inhibiting ribosome assembly and translation to enhance survival under nutrient limitation, osmotic stress, heat shock, and oxidative stress (53). Known as the “magic spot nucleotide,” ppGpps are alarmones involved in the stringent response in bacteria, which is triggered by a wide range of environmental stress conditions (54). Accumulation of (p)ppGpp represses the transcription of the *pur* operon, reducing purine biosynthesis (55). *De novo* purine synthesis consumes at least five ATP molecules, bacteria tend to suppress this pathway under energy-limited stress conditions and instead rely on purine salvage (10). Together, these mechanisms illustrate how LAB coordinate metabolic and translational regulation to maintain cellular homeostasis during oxidative stress.

Figure 6 illustrates genes specifically upregulated in the evolved strains relative to the wild type, which likely underpin their enhanced adaptability. Inactivating ATPase CopB increased *L. plantarum*’s sensitivity to H_2_O_2_, highlighting the role of metal ion transport in oxidative stress (49). In response to H_2_O_2_, *L. plantarum* upregulates *copB* to reduce intracellular copper levels, thereby enhancing oxidative resistance (56). Cell wall expansion is driven by the coordinated actions of peptidoglycan synthases and hydrolases and may also be regulated by central carbon metabolism (57). For example, *cwlO* is upregulated under prolonged acidic conditions, maintaining structural integrity through cell wall remodeling (58). A two-component system was significantly enriched, which is highly versatile in responding to diverse environmental stress signals, such as regulating cell wall synthesis and biofilm formation (59).

Aromatic amino acids, such as phenylalanine, tyrosine, and tryptophan, serve as precursors for antioxidant molecules, and their catabolism may enhance cellular adaptability to fluctuating oxygen levels (60). Gamma-glutamylleucine may alleviate oxidative stress via the glutathione cycle (61), and dipeptides such as these have demonstrated antioxidant properties, mitigating oxidative damage and inflammation (62). Consistent with previous findings (2), palmitic acid was significantly reduced in the evolved strain, likely reflecting altered membrane fatty acid composition to regulate fluidity and permeability (63). Conversely, nicotinic acid, a form of vitamin B3, was enriched in the evolved strain, contributing to antioxidant defense through increased glutathione and NAD levels (64). As a precursor for NAD^+^ biosynthesis, nicotinic acid enrichment suggests that the evolved strain enhances metabolic activity and redox regulation via increased NAD^+^ production. NOX and NADH peroxidase utilize NADH as an electron donor to neutralize ROS, while thioredoxin reductase employs NADPH to sustain the reduced state of thioredoxin (65).

The most pronounced gene expression differences were observed in citrate metabolism, which was consistently and significantly upregulated in the evolved strains (Fig. 6D). The overall alkalinizing effect of citrate catabolism may counteract the acidification caused by carbohydrate metabolism that inhibits bacterial growth (66). The transport and utilization of citrate generates proton motive force, thereby facilitating additional ATP production (67). This process supports the regeneration of NAD^+^ and NADPH, which in turn facilitates glutathione (GSH) recycling, GABA production, and redox homeostasis. Acetyl-CoA from citrate metabolism serves as a precursor for fatty acid biosynthesis, and upregulation of this pathway may enhance membrane repair and remodeling, maintaining integrity under oxidative stress (14, 68). By coordinating flux across multiple pathways, the pentose phosphate pathway (PPP) governs biosynthetic capacity under stress (69). Histidine, a conditionally essential amino acid during induced stress, possesses intrinsic antioxidant properties and supports other essential cellular functions (70).

**Fig 7:**
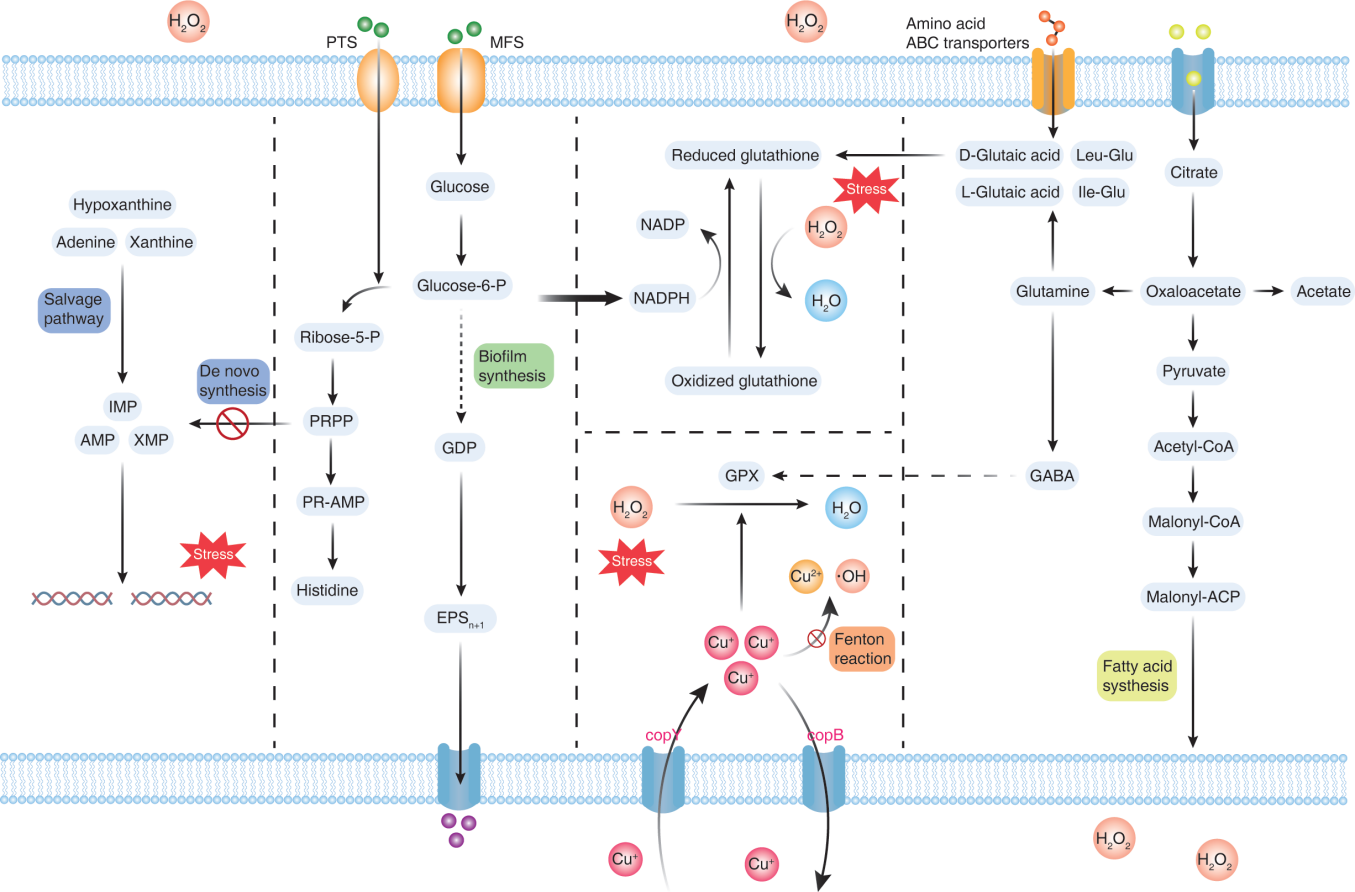
Mechanism of enhanced oxidative stress response in evolved *L. casei*. XMP: xanthosine monophosphate; IMP: inosine monophosphate; Glucose-6-P: glucose-6-phosphate; GDP: guanosine diphosphate; EPS_(n+1)_: exopolysaccharide with (*N* + 1) repeating units; GPX: glutathione peroxidase; GABA: gamma-aminobutyric acid.

A schematic diagram summarizes the potential mechanisms contributing to the enhanced oxidative stress tolerance observed in the evolved strains (Fig. 7). The evolved strains exhibited higher survival rates and greater biomass under oxidative stress conditions. These advantages are attributed to (i) an enhanced purine salvage pathway that supports the conserved stringent response in LAB. This pathway ensures sufficient production of IMP, GMP, and XMP for nucleic acid synthesis and DNA repair under stress conditions; (ii) upregulated transmembrane transport that enhances carbon source utilization and ATP production to sustain bacterial growth. In addition, histidine biosynthesis is promoted via the PRPP pathway; (iii) increased NADPH production provides reducing power to regenerate oxidized GSH, maintaining intracellular redox balance; (iv) regulation of metal ion homeostasis via transmembrane transport proteins, which suppresses Fenton reactions and minimizes the generation of highly toxic ROS; (v) finally, upregulation of citrate transport and degradation significantly increases pyruvate and acetate production. This may alter fatty acid composition, facilitating membrane remodeling.

In conclusion, the evolved strain primarily restructured metabolic pathways to improve carbon source utilization, particularly of citrate, thereby boosting ATP production and reducing power. Additionally, enhanced transmembrane transport helped maintain intracellular metal ion homeostasis, thereby suppressing the Fenton reaction and hydroxyl radical generation. These two key mechanisms jointly contributed to the enhanced oxidative stress tolerance of the evolved strain. This study avoided direct genetic manipulation of LAB and instead provides mechanistic insights for probiotic optimization in the food and health industries.

## Data Availability

The whole-genome sequence generated in this study has been deposited in the NCBI Bioproject database under accession number PRJNA1330167. Raw transcriptomic sequences were deposited in the NCBI Sequence Read Archive (SRA) under accession number PRJNA1254908.

## SUPPLEMENTAL MATERIAL

Supplemental figures aem.01854-25-s0001.docx Figures S1 to S3.

Supplemental tables aem.01854-25-s0002.xlsx Tables S1 to S10.

ASM does not own the copyrights to Supplemental Material that may be linked to, or accessed through, an article. The authors have granted ASM a non-exclusive, world-wide license to publish the Supplemental Material files. Please contact the corresponding author directly for reuse.
