# Supplementary material for: Adaptive metabolic rewiring and ion homeostasis enhance oxidative stress resistance in Lacticaseibacillus casei
Source: Appl Environ Microbiol. 2025 Dec 16;92(1):e01854-25. doi: 10.1128/aem.01854-25 (PMC12838381; doi:10.1128/aem.01854-25)
Supplement: Supplemental figures — Figures S1 to S3. [file aem.01854-25-s0001.docx]

**Adaptive metabolic rewiring and ion homeostasis enhance oxidative stress resistance in *Lacticaseibacillus casei***

*Lei Su^1,2^, Ruisi Yang^1,2^, Shan Li^1,2^, Qilin Zhang ^1,2^,Feng Wang^1,2^, Lianbing Lin^1,2 *^*, *Yicen Lin^1,2 *^*

*^1^ Faculty of Life Science and Technology, Kunming University of Science and Technology, Yunnan Kunming 650500, China*

*^2^ Engineering Research Center for Replacement Technology of Feed Antibiotics of Yunnan College, Yunnan, Kunming, 650500, China*

*Correspondence: [yicenlin@kust.edu.cn](mailto:yicenlin@kust.edu.cn); linlb@kust.edu.cn

**Figures**

**
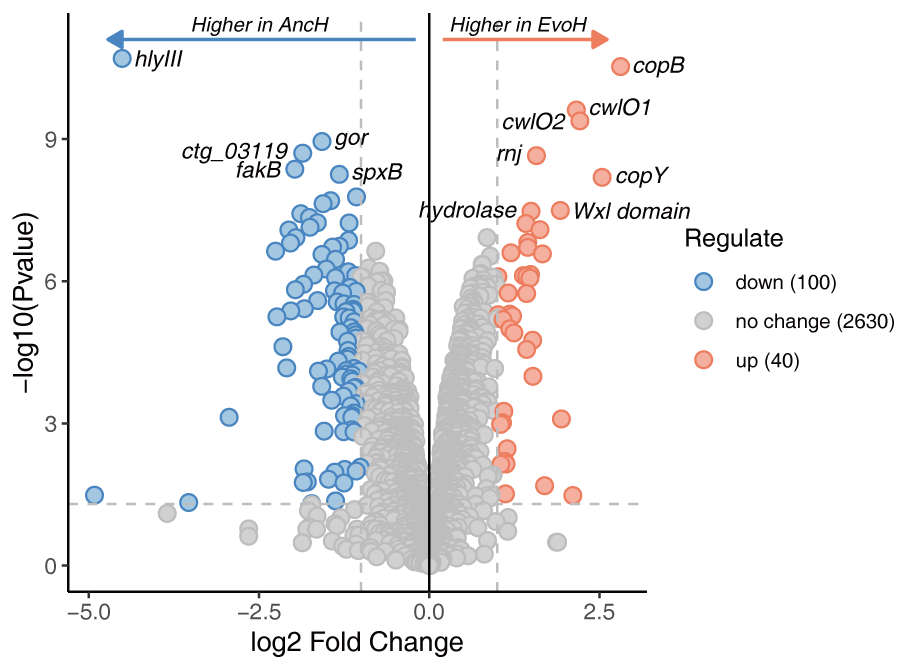
**

**Figure S1.** Volcano plot showing transcriptomic differences between EvoH and AncH.


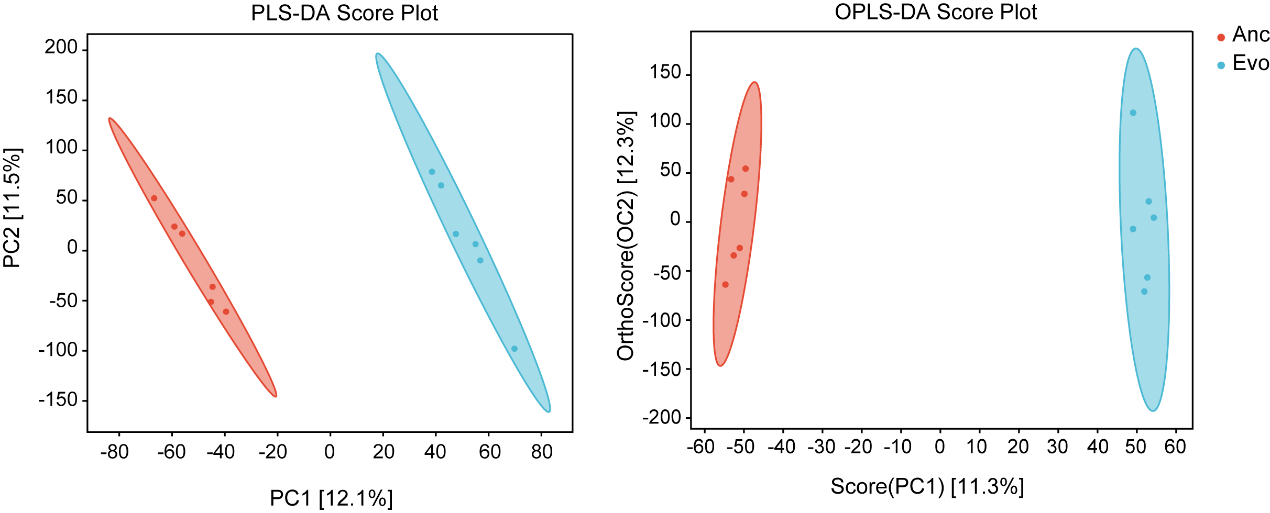


**Figure S2.** PLS-DA and OPLS-DA Score Plots.


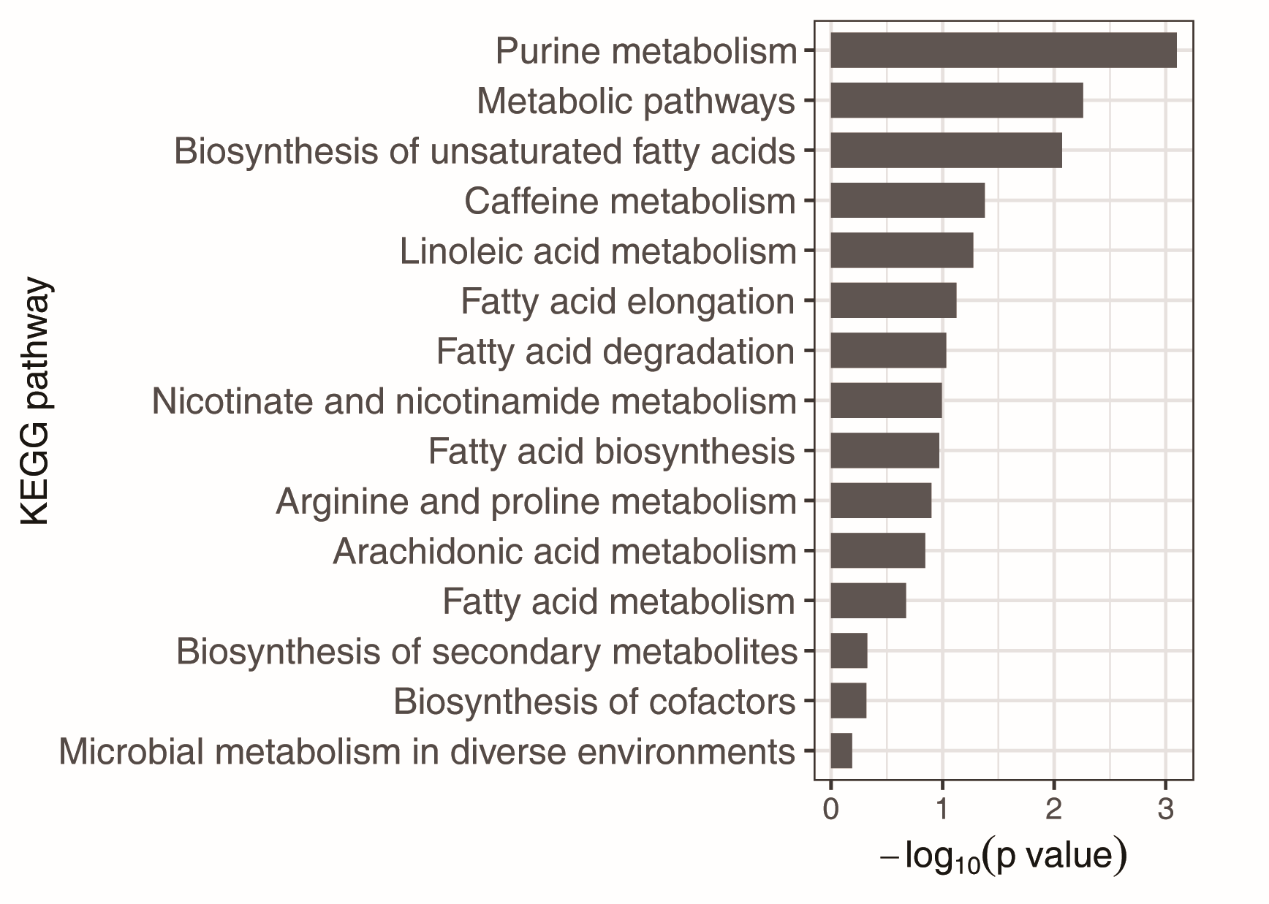


**Figure S3.** KEGG pathway maps of Anc and Evo strains.
